# Retention on ART and viral suppression among patients in alternative models of differentiated HIV service delivery in KwaZulu-Natal, South Africa

**DOI:** 10.1371/journal.pgph.0000336

**Published:** 2022-12-14

**Authors:** Altynay Shigayeva, Ntombi Gcwensa, Celiwe Dlamini Ndlovu, Nosicelo Ntumase, Scelinhlanhla Sabela, Liesbet Ohler, Laura Trivino-Duran, Ellie Ford Kamara, Khanyo Hlophe, Petros Isaakidis, Gilles Van Cutsem

**Affiliations:** 1 Médecins Sans Frontières—South Africa, Eshowe, South Africa; 2 Médecins Sans Frontières—South Africa, Cape Town, South Africa; 3 King Cetshwayo District Department of Health; 4 Southern African Medical Unit, Médecins Sans Frontières, Cape Town, South Africa; 5 Clinical and Molecular Epidemiology Unit, Department of Hygiene and Epidemiology, University of Ioannina School of Medicine, Ioannina, Greece; 6 Centre for Infectious Disease Epidemiology and Research, University of Cape Town, South Africa; University of California San Francisco, UNITED STATES

## Abstract

Differentiated models of HIV care (DMOC) aim to improve health care efficiency. We describe outcomes of five DMOC in KwaZulu-Natal, South Africa: facility adherence clubs (facility AC) and community adherence clubs (community AC), community antiretroviral treatment (ART) groups (CAG), spaced fast lane appointments (SFLA), and community pick up points (PuP). This retrospective cohort study included 8241 eligible patients enrolled into DMOC between 1/1/2012 and 31/12/2018. We assessed retention in DMOC and on ART, and viral load suppression (<1000 copies/mL). Kaplan-Meier techniques were applied to describe crude retention. Mixed effects parametric survival models with Weibull distribution and clustering on health center and individual levels were used to assess predictors for ART and DMOC attrition, and VL rebound (≥1000 copies/mL). Overall DMOC retention was 85%, 80%, and 76% at 12, 24 and 36 months. ART retention at 12, 24 and 36 months was 96%, 93%, 90%. Overall incidence rate of VL rebound was 1.9 episodes per 100 person-years. VL rebound rate was 4.9 episodes per 100 person-years among those enrolled in 2012–2015, and 0.8 episodes per 100 person-years among those enrolled in 2016–2018 (RR 0.12; 95% CI, 0.09–0.15, p<0.001). Prevalence of confirmed virological failure was 0.6% (38/6113). Predictors of attrition from DMOC and from ART were male gender, younger age, shorter duration on ART before enrollment. Low level viremia (>200–399 copies/mL) was associated with higher hazards of VL rebound and attrition from ART. Concurrent implementation of several DMOC in a large ART program is feasible and can achieve sustained retention on ART and VL suppression.

## Introduction

During the last decade, several countries in sub-Saharan Africa introduced differentiated models of HIV care (DMOC), to address the needs of patients and reduce unnecessary burdens on the health system [1]. DMOC offer patients antiretroviral treatment (ART) services that allow them to pick up medication in a less time consuming manner and/or in a convenient community location, obtain counseling and peer support, and reduce the number of visits to a clinic [2,3]. Compared to clinic-based care, DMOC were shown to have comparable or better outcomes in retaining patients on ART and/or maintaining viral suppression. Examples include community ART groups [4,5], adherence clubs [3,6–8], six months refill models [9,10], and community distribution points [11,12]. Qualitative research demonstrated that DMOC were generally acceptable for patients, who benefited from peer support, reduced waiting times or visit frequency [13,14].

Sustainability remains a concern as implementation of DMOC at scale requires financial and human resources in order to ensure continuous clinical monitoring and counseling [2,15]. Patients’ preferences and the support they require change over time [16], thus identifying the optimal combination of care models that are responsive to patients’ needs has been among the challenges [2,17]. There is limited published evidence on concurrent implementation of different models, including their long-term outcomes [18]. This study describes DMOC that were implemented under routine programmatic conditions in KwaZulu-Natal, South Africa. Between 2012 and 2016, five DMOC were initiated: facility adherence clubs (facility AC), community adherence clubs (community AC), community ART groups (CAG), spaced fast lane appointments (SFLA), and decentralized medication delivery at community pick up points (PuP). We compare retention in DMOC, retention on ART and viral load (VL) suppression among patients who received HIV care in these five different DMOC models.

## Methods

### Study settings

Since 2011 Médecins Sans Frontières (MSF) and the Department of Health (DoH) of KwaZulu-Natal have been implementing the community based “*Bending the Curves*” HIV/TB project [19]. The project involves two hospitals and ten health centres in the Mbongolwane and Eshowe areas, covering a population of around 125,000. In 2018, HIV prevalence in the study area was an estimated 26.4% among 15–59 years old adults [20].

The DMOC program was initiated in 2012 with the introduction of counselor–led facility adherence AC and CAG. Adherence clubs (AC) are groups of 20–30 patients who meet every 2–3 months with a lay counselor to receive pre-packed medications, adherence counseling and peer support, either at a health facility (facility AC) or at a patient’s house or public venue (community AC). CAG are peer support groups of 4–6 patients, in which patients self-organize and take turns where one patient picks up medicines for the whole group. In 2014, the National Department of Health (NdoH) of South Africa adopted the Central Chronic Medicines Dispensing and Distribution (CCMDD) program in its National Adherence Guidelines for TB, HIV and NCDs (AGLs) [21] to improve the access to ART and other chronic disease medications via community, pharmacy or clinic-based pick up sites. Consequently, the DMOC program in our area was expanded in 2016 with the introduction of two individual models, SFLA and PuP. SFLA allows patients to receive multi-month prescriptions (6 months) and visit a clinic only for refills (2–3 months). Within PuP, patients receive medicines at pick-up sites in a community location. Patients in SFLA and PuP have spaced clinical visits, 6-monthly or annually, for clinical monitoring.

Implementation of DMOC occurred gradually, with facility AC starting in 2012, CAG in 2013, community AC in 2014, SFLA in 2015, and finally PuP in 2016. Rapid scale up of SFLA and PuP started in 2016, as a part of overall CCMDD program roll out in the country [22]

Eligibility criteria for DMOC included: age ≥18 years, being on the same ART regimen for at least 12 months, having a viral load (VL) in past 6 months, and two last VL <400 copies/ml, without active tuberculosis (TB), pregnancy, or other conditions requiring regular clinical consultations [21]. Patients who were enrolled into DMOC could return to standard clinic care (SoC) either for personal reasons, pregnancy, or clinical reasons. If they missed an appointment or prescription pick-up for more than 30 days [21], DMOC patients would be returned to SoC on administrative grounds.

### Study design

This retrospective cohort study compared outcomes of patients enrolled into each of the five DMOC models (facility AC, community AC, CAG, SFLA and PuP) in 10 health centers in the Eshowe and Mbongolwane areas. We included in the analysis patients who were initially enrolled into a DMOC between 1 January 2012 and 31 December 2018. Patients who started DMOC without meeting the eligibility criteria [21] were excluded.

Patients were identified and followed using routinely collected data. The main data source was TIER.net, the national electronic register system for HIV and TB programs [23].

### Data management

The dataset for this study was prepared by extracting data from TIER.net, followed by data verification and clean up. TIER.net collects demographic, clinical and outcome data for every patient registered for ART at a health facility. Additionally, TIER.net captures patients’ visit to receive ART, including date, prescribed ART regimen, date of next appointment, and provider of ART. The later field (provider of ART) records SoC or type of DMOC (e.g. CAG, SFLA, PuP). MSF provided technical assistance to health centers in setting up data capture of DMOC visits into TIER.net (details are outlined in S1 File). TIER.net does not contain the date of enrollment or the date of exit from DMOC (there are no dedicated fields). However, information on enrollment into or exit from DMOC was routinely recorded in patients’ files and facility based DMOC registers.

DMOC visits information was assessed for accuracy and completeness to assure key visits such as the first visit (enrolment) and last DMOC visit were captured. For this analysis, the enrollment date into a DMOC was defined as the first documented DMOC visit.

We identified patients whose DMOC information required verification: a) patients who were enrolled in DMOC but were not meeting eligibility criteria; b) patients whose DMOC visits were not updated; c) patients who were recorded in registers as actively in DMOC but whose DMOC visits were not entered in TIER.net, d) patients who were listed in a facility-based register as a DMOC participant but not indicated in TIER.net as such.

DMOC visit information was verified using patient files, facility AC and CAG registers, SFLA log books (available at some facilities), and Centralized Chronic Medicine Dispensing and Distribution (CCMDD) registers (both paper-based and electronic since 2019). CCMDD registers document medication distribution at the pick-up points. We verified active CCMDD participants by linking CCMDD electronic data with TIER.net data through matching of personal identification information such surname, name, date of birth, and ART file number. Additionally, data was crossed checked manually through review of patients’ files.

### Measures and definitions

Baseline characteristics at the time of DMOC initiation included age, last known CD4 count, VL, ART regimen, and time on ART at enrollment. Patients’ age was grouped as 18–29, 30–39, and ≥40 years old. We categorised CD4 count into <200 and ≥200 cells/μL and prior VL into <50, 50 to 199, and 200 to 399 copies/ml. VL ≥50 copies/mL and <400 copies/mL were referred as low-level viremia (LLV).

First line ART included regimens consisting of two nucleoside reverse transcriptase inhibitors (NRTI) and a non-nucleoside reverse transcriptase inhibitor (NNRTI) as recommended by national guidelines [24,25]. In our cohort, first line regimens included either stavudine, tenovofir or zidovudine combined with lamivudine as NRTI-backbone, with efavirenz or nevirapine as the NNRTI. Second line ART regimens included the ritonavir-boosted protease inhibitor (PI) lopinavir-ritonavir (LPVr) combined with two NRTIs.

We define two implementation periods, 2012–2015 and 2016–2018, to account for major changes in the DMOC program, coinciding with the rapid scale up of SFLA and PuP initiated in 2016.

### Outcomes

We assessed three outcomes: retention in DMOC, retention on ART and VL suppression.

Retention in a DMOC was defined as the time from DMOC enrollment to the composite endpoint of death, loss to follow up (LTFU), or exit from DMOC to return to SoC clinic. In case of return, to SoC, date of return was the date of last DMOC visit. LTFU was defined as > = 90 days since last missed appointment to receive ART medicines; date of LTFU was the date of the last visit with a provider. If a patient changed between DMOC models, the follow up was censored on the date of transfer to the other model. For retention on ART, the outcome was the time from DMOC enrollment to the endpoint of death or LTFU, irrespective of whether the event occurred whilst in DMOC or upon return to SoC. For all analyses, censoring occurred in case of transfer out to other health facilities (non-study), or database closure on 31/12/2019.

VL suppression was defined as VL <1000 copies/ml. VL rebound was defined as a VL ≥1000 copies/ml after enrollment into a DMOC (baseline at enrollment was VL <400 copies/ml). Virological failure (VF) was defined as two consecutive VL measurements ≥1000 copies/ml (taken two to twelve months apart). Date of second elevated VL was considered as a date of VF. For VL rebound, patients were followed from the date of DMOC enrollment until the date of VL rebound or censored on the date of last DMOC visit. Patients who had at least one VL test after DMOC enrollment were included into the VL rebound analysis.

### Statistical analysis

Patient characteristics were summarized as median and interquartile ranges for continuous variables, and as frequencies and proportions for categorical variables. Characteristics were compared using Chi-square or Fisher’s exact tests for categorical variables, and Kruskal Wallis tests for continuous variables.

Kaplan-Meier techniques were applied to describe retention in DMOC and on ART. Log rank test was applied to assess differences in survival distributions among care models. For Kaplan Meier estimates, the follow up was restricted to the initial DMOC (i.e., changes in DMOC were not accounted for).

We applied mixed-effects parametric survival models to estimate hazard ratios (HR) for factors associated with attrition from DMOC, attrition from ART, and VL rebound. To account for changes in DMOC types during the follow up, DMOC was modelled as a time varying exposure variable. We fitted models with Weibull, log-normal and loglogistic distributions, and evaluated models using Akaike Information Criterion (AIC) [26]. The models were fitted with health center level random intercept, and individuals nested within health centers (i.e., adjusted for clustering at health center and an individual levels). The STATA “mestreg” command was used for the estimations [27]. Models with Weibull as a baseline distribution had the lowest AIC and were selected as final models.

Predictors tested in the models included: year of enrollment into a DMOC model, type of DMOC and personal characteristics at time of DMOC enrollment: age, gender, time on ART, last known CD4 count and VL, and ART regimen.

Data were cleaned, coded and analyzed using STATA version 16 (College Station, TX; StataCorp). In all analyses, *p-*value ≤0.05 was considered statistically significant.

### Ethics statement

Ethical approval was received from the University of Cape Town Human Research Ethics Committee (HREC REF 036/2012), the Provincial Health Research Unit of the KwaZulu Natal Department of Health (HRKM104/12), and the Médecins Sans Frontières Ethics Review Board (Reference: ID 1204). As this was a study of routinely collected monitoring data, informed consent from the participants was not obtained. The named ethics committees waived the need for consent.

## Results

**Fig 1:**
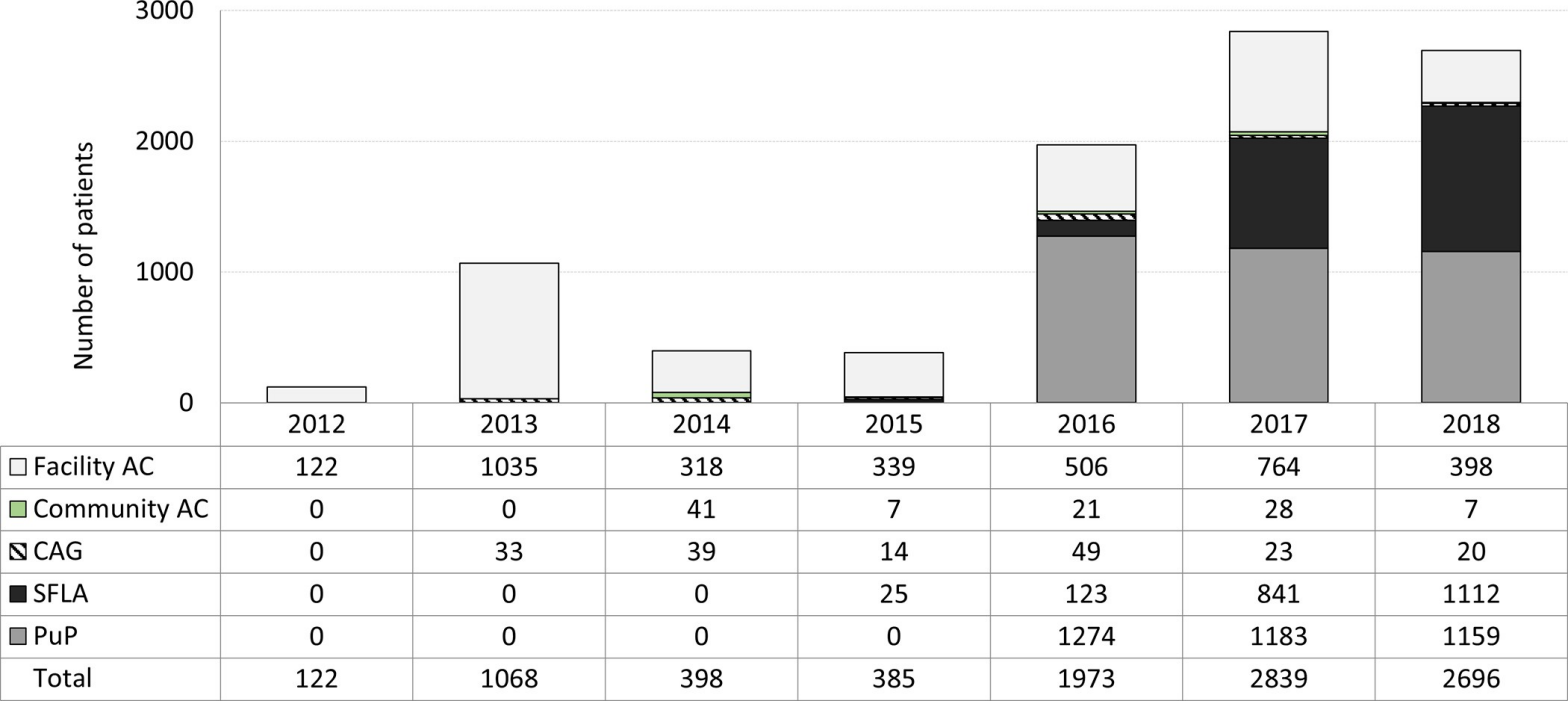
Annual enrollment of new patients into DMOC, by type of model, 2012–2018, KwaZulu-Natal, South Africa. Stacked bar char, x-axis = year: *the white* sub-bar = facility AC, facility adherence club; *the green sub-bar* = community AC, community adherence club; *hatched* sub-bar = CAG, community ART group; *the black* sub-bar = SFLA, spaced and fast lane appointment; *the grey* sub-bar = PuP, decentralized medication delivery at community pick up points.

Between 2012 and 2018, 9481 patients were enrolled in DMOC (Fig 1). Between 2012 and 2015, 1973 patients were enrolled, 91.9% (1814/1973) of whom in facility AC. In the 2016–2018 period, 7508 patients were enrolled, with average annual enrollment at 2500 persons per year. Initially the majority were in PuP (64% in 2016, 42% in 2017/2018), with a gradual increase of SFLA to over 41% of enrolments in 2018. Enrolments in CAG and community AC remained low, accounting for an average of 4.1% of new DMOC participants in the first study period and 1.4% in the second study period. 1240/9481 (13%) of enrolled patients didn’t meet DMOC eligibility criteria and were excluded from the analysis.

### Patient characteristics at time of dmoc enrollment

Among 8241 included DMOC patients (Table 1), 2721 (33%) were enrolled in facility AC, 81 (1.0%) in community AC, 133 (1.6%) in CAG, 1973 (23.9%) in SFLA, and 3333 (40.4%) in PuP. Median age among DMOC patients was 39.4 years (IQR 32.4–48.1). Patients in community AC were older (median 44.7 years old) and in PuP were younger (median 38.1 years old) than participants in other models (*p* = 0.001). Overall, 2048 (24.8%) were men, with a higher proportion of men in community AC (34.6%), followed by SFLA (29.2%) and PuP (26%, *p* = 0.008). The last known CD4 count prior DMOC enrollment was a median 520 cells/μL (IQR 376–690); the lowest median CD4 count was among community AC patients (median 450 cells/μL), and highest among PuP (median 545 cells/μL, *p*<0.001). Time on ART prior to enrollment was 3.8 years (IQR 2.2–6.2); the time was lower among facility AC (median 3.2 years) and higher among SFLA participants (median 5 years; *p*<0.001). 415 (5%) were on second line ART at time of DMOC enrollment; the higher proportions of patients on second line were among community AC participants (11%;), followed by CAG (8%) and SFLA (7%, *p*<0.001).

**Table 1:** Baseline characteristics and outcomes of patients enrolled in DMOC in 2012–2018, by initial DMOC type.

	Facility AC	Community AC	CAG	SFLA	PuP	DMOC (all models)
	N = 2721	N = 81	N = 133	N = 1973	N = 3333	N = 8241
	N (%)	N (%)	N (%)	N (%)	N (%)	N (%)
Gender						
Female	2168 (79.7)	53 (65.4)	108 (81.2)	1397 (70.8)	2467 (74.0)	6193 (75.1)
Male	553 (20.3)	28 (34.6)	25 (18.8)	576 (29.2)	866 (26.0)	2048 (24.9)
Age group						
18–29 years old	456 (16.8)	6 (7.4)	25 (18.8)	276 (14.0)	673 (20.2)	1436 (17.4)
30–39 years old	934 (34.3)	21 (25.9)	42 (31.6)	671 (34.0)	1161 (34.8)	2829 (34.3)
≥ 40 years old	1331 (48.9)	54 (66.7)	66 (49.6)	1026 (52.0)	1499 (45.0)	3976 (48.2)
Median (IQR), in years	39.6 (32.4–48.3)	44.7 (37.5–54.9)	39.8 (31.6–48.1)	40.6 (33.7–48.9)	38.1 (31.5–47.4)	39.4 (32.4–48.1)
Time on ART ^1^						
Median (IQR), in years	3.2 (1.7–5.4)	3.9 (1.9–6.1)	3.6 (2.5–5.3)	5.0 (3.1–7.2)	3.6 (2.1–5.9)	3.8 (2.2–6.2)
CD4 group ^2^, cells/μL						
≥ 200	2550 (93.7)	70 (86.4)	128 (96.2)	1870 (94.8)	3210 (96.3)	7828 (95.0)
<200	160 (5.9)	10 (12.3)	5 (3.8)	91 (4.6)	113 (3.4)	379 (4.6)
Not done	11 (0.4)	1 (1.2)	0 (0.0)	12 (0.6)	10 (0.3)	34 (0.4)
Median (IQR), cells/μL	494 (355–665)	450 (312–640)	522 (386–672)	513 (366–691)	545 (403–708)	520 (376–690)
VL group ^2^ (copies/ml)						
< 50	2192 (80.6)	52 (64.2)	107 (80.5)	1801 (91.3)	3005 (90.2)	7157 (86.8)
50–199	366 (13.5)	21 (25.9)	21 (15.8)	147 (7.5)	283 (8.5)	838 (10.2)
200–399	163 (6.0)	8 (9.9)	5 (3.8)	25 (1.3)	45 (1.4)	246 (3.0)
ART regimen on enrollment						
First Line ART	2599 (95.5)	72 (88.9)	122 (91.7)	1832 (92.9)	3201 (96.0)	7826 (95.0)
Second line ART	122 (4.5)	9 (11.1)	11 (8.3)	141 (7.1)	132 (4.0)	415 (5.0)
Changed DMOC model	866 (31.8)	15 (18.5)	44 (33.1)	140 (7.1)	481 (14.4)	1546 (18.8)
DMOC outcome						
Remain in DMOC ^3^	2072 (76.1)	51 (63.0)	82 (61.7)	1369 (69.4)	2455 (73.7)	6029 (73.2)
Returned to SoC	349 (12.8)	22 (27.2)	31 (23.3)	268 (13.6)	401 (12.0)	1071 (13.0)
LTFU	141 (5.2)	7 (8.6)	12 (9.0)	75 (3.8)	237 (7.1)	472 (5.7)
Died	34 (1.2)	0 (0.0)	4 (3.0)	6 (0.3)	20 (0.6)	64 (0.8)
Transferred out	125 (4.6)	1 (1.2)	4 (3.0)	255 (12.9)	220 (6.6)	605 (7.3)
ART outcome						
Active on ART	2255 (82.9)	70 (86.4)	105 (78.9)	1608 (81.5)	2784 (83.5)	6822 (82.8)
LTFU	212 (7.8)	9 (11.1)	14 (10.5)	87 (4.4)	262 (7.9)	584 (7.1)
Died	49 (1.8)	1 (1.2)	5 (3.8)	8 (0.4)	23 (0.7)	86 (1.0)
Transferred out	205 (7.5)	1 (1.2)	9 (6.8)	270 (13.7)	264 (7.9)	749 (9.1)

^1^ Time on ART prior the date of enrollment into DMOC

^2^ Last known measure prior the date of DMOC enrollment; for CD4, “Not done” includes observations that were not done or missing.

^3^ If individual changed DMOC type, considered as remained in DMOC

Abbreviations: DMOC, differentiated model of HIV care; CAG, community ART group; facility AC, facility adherence club; community AC, community adherence club; SFLA, spaced and fast lane appointment; PuP, decentralized medication delivery at community pick up points; SoC, standard of care; ART, antiretroviral therapy; VL, viral load; LTFU, lost to follow up

### Retention in DMOC and factors associated with all-cause attrition

Out of 8241 DMOC patients, 1071 (13.2%) returned to SoC, 472 (5.8%) were LTFU, 64 (0.8%) died (Table 1). 1546/8241 (18.8%) patients changed their DMOC type, with highest proportion observed among those who were initially enrolled in facility AC (31.8%) and CAG (33.1%) (Table 1). Out of 1546 who moved, the most frequent moves were into PuP (39.2%, N = 606) and SFLA (41.8%, N = 647), followed by moves to facility AC (13.3%, N = 205), CAG (3.9%, N = 60) and community AC (1.8%, N = 28). As of last DMOC visit, 2060 (25%) received care in facility AC, 94 (1.1%) in community AC, 149 (1.8%) in CAG, 2480 (30.1%) in SFLA, and 3458 (42%) in PuP.

**Fig 2:**
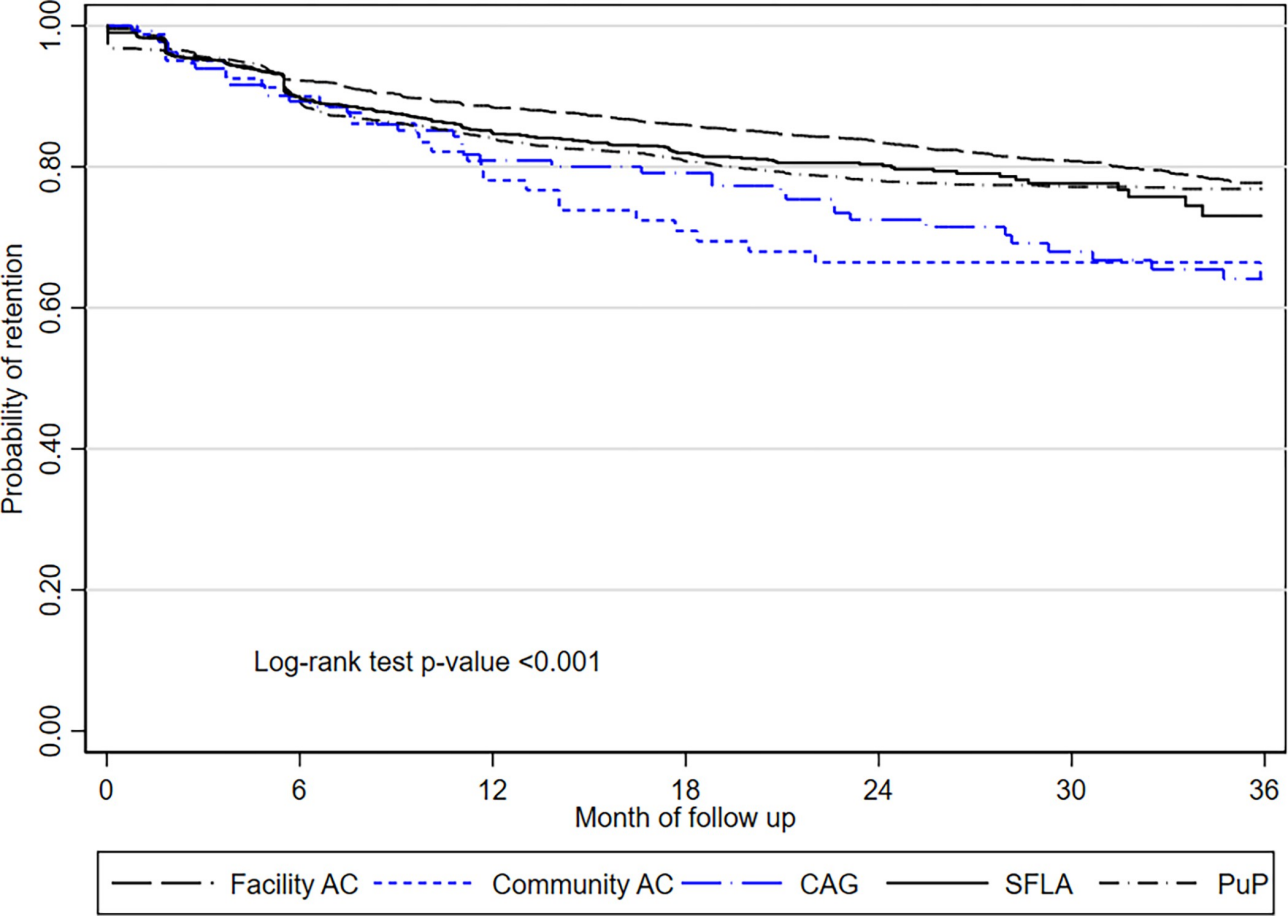
Kaplan Meier curves for retention in DMOC, by type of model, KwaZulu-Natal, South Africa. *The* *long dash black line* = facility AC, facility adherence club; *the short dash blue line* = community AC, community adherence club; *the long dash-dot blue line* = CAG, community ART group; *the solid black line =* SFLA, spaced and fast lane appointment model; *the dash-dot black line*, PuP = decentralized medication delivery at community pick up points.

Overall DMOC retention was 85%, 80%, and 76% at 12, 24 and 36 months (Fig 2). DMOC retention was higher at facility AC (88%, 83%, 78% at 12, 24 and 36 months), and lower for CAG (81%, 73%, and 64%) and community AC (77%, 65%, 63%) as compared to other models (*p* = 0.003). Retention among PuP (84%, 78%, 77% at 12, 24 and 36 months) and SFLA participants (85%, 80%, 73%) did not differ (*p* = 0.4) but was lower as compared to facility AC (*p* = 0.003).

Overall annual all-cause DMOC attrition rate was 10.6 per 100 person-years during the study period. Attrition rate was 15.9 per 100 person-years in the first year, 6.7 and 4.9 per 100 person-years during the second and third year of follow up, respectively. Annual rate of attrition was 7.5 per 100 person-years among DMOC patients enrolled in 2012–2015, and 11.9 per 100 person-years among those enrolled in 2016–2018 (2016–2018 vs 2012–2015: RR 1.2; 95% CI, 1.03–1.4, *p* = 0.01).

In the multivariate survival model (Table 2), factors associated with all-cause DMOC attrition (LTFU, death or return to SoC) were age (18–29 years; aHR 1.7; 95% CI, 1.5–2.0 and ≥40 years; aHR 0.8; 95% CI, 0.7–0.9), being male (aHR 1.3; 95% CI, 1.2–1.5), initiating DMOC whilst on 2^nd^ line ART (aHR 1.4; 95% CI, 1.1–1.8). Patients who were more experienced on ART prior to joining a DMOC were less likely to leave the model (one-year increase: aHR 0.97; 95% CI, 0.95–0.99). Relative to facility AC, patients in PuP (aHR 1.2; 95% CI, 1.0–1.4). and CAG (aHR 1.5; 95% CI, 1.0–2.1) were more likely to leave DMOC. Year of DMOC enrollment was associated with increased hazards of attrition (one year increase: aHR 1.1; 95%, 1.0–1.1).

**Table 2:** Weibull parametric survival model^1^ for factors associated with all cause attrition from DMOC (death, LTFU or return to clinic care).

	Univariate analysis		Multivariate analysis	
	HR (95% CI)	*p-*value	aHR (95% CI)	*p-*value
Gender				
Female	Reference		Reference	
Male	1.22 (1.08–1.38)	0.001	1.32 (1.17–1.50)	<0.001
Age group				
18–29 years old	1.70 (1.46–1.98)	<0.001	1.72 (1.47–2.01)	<0.001
30–39 years old	Reference		Reference	
≥ 40 years old	0.79 (0.70–0.90)	<0.001	0.80 (0.70–0.90)	<0.001
CD4 group^2^, cells/μL				
≥ 200	Reference		Reference	
<200	1.27 (0.99–1.61)	0.06	1.22 (0.96–1.56)	0.10
Not done	0.87 (0.35–2.14)	0.76	0.84 (0.35–2.04)	0.7
VL group^2^, copies/ml				
<50	Reference		Reference	
50–199	0.98 (0.82–1.17)	0.84	1.01 (0.85–1.20)	0.92
200–399	1.07 (0.81–1.42)	0.62	1.26 (0.95–1.67)	0.10
Time on ART prior enrollment (1 year increase)	0.95 (0.93–0.97)	<0.001	0.97 (0.94–0.99)	0.01
ART regimen on enrollment				
First Line ART	Reference		Reference	
Second line ART	1.19 (0.93–1.51)	0.16	1.38 (1.07–1.77)	0.01
Model type				
Facility AC	Reference		Reference	
Community AC	1.36 (0.88–2.10)	0.16	1.34 (0.87–2.07)	0.18
CAG	1.46 (1.04–2.05)	0.03	1.46 (1.04–2.05)	0.03
SFLA	1.24 (1.05–1.46)	0.01	1.16 (0.96–1.40)	0.11
PuP	1.30 (1.14–1.49)	<0.001	1.21 (1.03–1.40)	0.02
Year of DMOC enrollment^3^	1.08 (1.04–1.12)	<0.001	1.05 (1.00–1.10)	0.05

^1^ Weibull model with adjustment for clustering at health center and an individual levels

^2^ Last known measure prior DMOC enrollment

^3^ Year of enrollment into a DMOC model, 2012–2013 as a reference, 1 year increase

Abbreviations: DMOC, differentiated model of HIV care; CAG, community ART group; AC, adherence club; SFLA, spaced and fast lane appointment model; PuP, decentralized medication delivery at community pick up points; ART, antiretroviral therapy; VL, viral load; HR, hazard ratio; aHR, adjusted hazard ratio; CI, confidence interval.

### Retention on ART

**Fig 3:**
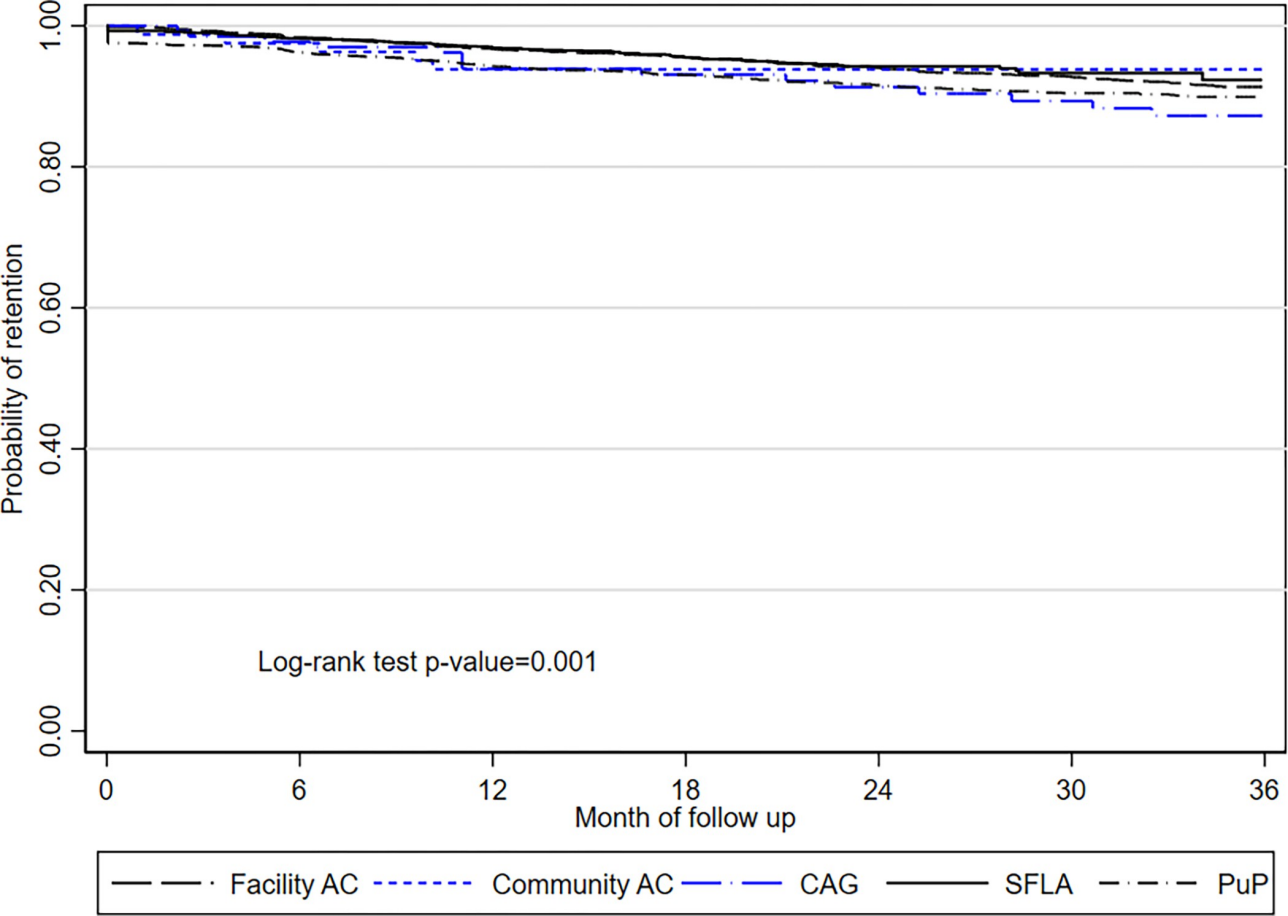
Kaplan Meier survival curves for probability of retention on ART among patients in DMOC, 2012–2018, KwaZulu Natal, South Africa. *The long dash black line* = facility AC, facility adherence club; *the short dash blue line* = community AC, community adherence club; *the long dash-dot blue line* = CAG, community ART group; *the solid black line =* SFLA, spaced and fast lane appointment model; *the dash-dot black line*, PuP = decentralized medication delivery at community pick up points.

ART retention at 12, 24 ad 36 months was 96%, 93%, 90% among DMOC patients overall (Fig 3). ART retention was highest among SFLA participants (97%, 94%, 92% at 12, 24 and 36 months), and facility AC (97%, 94% and 91%), which did not differ between both types (*p* = 0.53). Retention among PuP (94%, 92%, 90% at 12, 24, and 36 months), community AC (93%, 93%, 93%) and CAG participants (94%, 91%, 87%) did not differ (p = 0.4) but was lower as compared to SFLA (*p* = .006).

Factors associated with all-cause attrition from ART (death or LTFU) were (Table 3): age (18–29 years: aHR 1.6; 95% CI, 1.4–2.0, and ≥40 years: aHR 0.83; 95% CI, 0.7–0.9), being male (aHR 1.6; 95% CI, 1.4–1.9), and time on ART prior joining DMOC (per one year increase: aHR 0.92; 95% CI, 0.90–0.95). As compared to facility AC, attrition from ART was higher among PuP participants (aHR 1.3; 95% CI, 1.0–1.6), and did not differ for other types. Year of DMOC enrollment was associated with reduced hazards of attrition (one year increase: aHR 0.94; 95%, 0.88–1.0).

**Table 3:** Weibull parametric survival model^1^ factors associated with all cause attrition from ART (death or LTFU) among DMOC patients.

	Univariate analysis		Multivariate analysis	
	HR (95% CI)	*p-*value	aHR (95% CI)	*p-*value
Gender				
Female	Reference		Reference	
Male	1.47 (1.26–1.72)	<0.001	1.64 (1.39–1.92)	<0.001
Age group				
18–29 years old	1.62 (1.34–1.96)	<0.001	1.63 (1.35–1.98)	<0.001
30–39 years old	Reference		Reference	
≥ 40 years old	0.81 (0.68–0.96)	0.01	0.83 (0.70–0.98)	0.03
CD4 group^2^, cells/μL				
≥ 200	Reference		Reference	
<200	1.25 (0.92–1.70)	0.16	1.03 (0.75–1.42)	0.83
Not done	0.69 (0.17–2.76)	0.59	0.61 (0.15–2.45)	0.48
VL group^2^, copies/ml				
<50	Reference		Reference	
50–199	1.20 (0.97–1.48)	0.10	1.11 (0.90–1.38)	0.33
200–399	1.05 (0.74–1.51)	0.77	1.01 (0.70–1.45)	0.97
Time on ART prior enrollment (1 year increase)	0.89 (0.86–0.93)	<0.001	0.92 (0.89–0.95)	<0.001
ART regimen on enrollment				
First Line ART	Reference		Reference	
Second line ART	1.00 (0.71–1.40)	0.99	1.37 (0.96–1.94)	0.08
Model type				
Facility AC	Reference		Reference	
Community AC	1.36 (0.77–2.40)	0.28	1.44 (0.81–2.54)	0.21
CAG	1.37 (0.88–2.12)	0.17	1.41 (0.91–2.20)	0.13
SFLA	0.69 (0.55–0.88)	0.003	0.85 (0.65–1.12)	0.25
PuP	1.10 (0.92–1.30)	0.29	1.25 (1.02–1.55)	0.03
Year of DMOC enrollment^3^	0.95 (0.90–0.99)	0.02	0.94 (0.88–1.00)	0.04

^1^ Weibull model with adjustment for clustering at health center and an individual levels

^2^ Last known measure prior DMOC enrollment

^3^ Year of enrollment into a DMOC model, 2012–2013 as a reference, 1 year increase

Abbreviations: DMOC, differentiated model of HIV care; CAG, community ART group; AC, adherence club; SFLA, spaced and fast lane appointment model; PuP, decentralized medication delivery at community pick up points; SoC, standard of care; ART, antiretroviral therapy; VL, viral load; HR, hazard ratio; aHR, adjusted hazard ratio; CI, confidence interval

### VL suppression

6113/8241 (74.2%) DMOC patients had at least one VL in the follow-up period. Elevated VL ≥1000 copies/mL was documented among 4.4% (272/6113) DMOC patients. 244/272 with VL rebound had confirmatory VL test, and VF was confirmed among 15.6% (38/244). Overall prevalence of VF was during the study period was 0.6% (38/6113).

Overall incidence rate of VL rebound was 1.9 episodes per 100 person-years; rate was 2.9 per 100 person-years in the first year of follow up, and 1.7 and 0.8 per 100 person-years the second and third year of follow up, respectively. VL rebound rate was 4.9 episodes per 100 person-years among those enrolled in 2012–2015, and 0.8 episodes per 100 person-years among those enrolled in 2016–2018 (RR 0.12; 95% CI, 0.09–0.15, p<0.00).

Factors associated with VL rebound (Table 4) were younger age (18–29 years, aHR 2.8 95% CI, 1.4–5.6), having LVL (for 200–399 copies/mL, aHR 3.1; 95% CI, 1.2–8.3), receiving second line ART (aHR 3.9; 95% CI, 1.6–9.8), and time on ART prior to joining DMOC (per one year increase; aHR 1.1; 95% CI, 1.0–1.2). As compared to facility AC, VL rebound did not differ for all types with exception of PuP participants (aHR 0.4; 95% CI, 0.2–0.8), and did not differ. Hazards of VL rebound decreased over the course of the study (1 year increase in DMOC enrollment aHR 0.4; 95% CI, 0.3–0.4).

**Table 4:** Weibull parametric survival model^1^ for factors associated with viral load rebound (> = 1000 copies/mL) among DMOC patients.

	Univariate analysis		Multivariate analysis	
	HR (95% CI)	*p-*value	aHR (95% CI)	*p-*value
Gender				
Female	Reference		Reference	
Male	0.75 (0.28–2.00)	0.56	1.15 (0.69–1.94)	0.59
Age group				
18–29 years old	1.59 (0.47–5.40)	0.46	2.80 (1.40–5.58)	0.003
30–39 years old	Reference		Reference	
≥ 40 years old	3.38 (1.38–8.27)	0.008	1.17 (0.70–1.95)	0.56
CD4 group^2^, cells/μL				
≥ 200	Reference		Reference	
<200	1.58 (0.22–11.48)	0.65	0.90 (0.28–2.85)	0.85
VL group^2^, copies/ml				
<50	Reference		Reference	
50–199	8.19 (3.41–19.67)	<0.001	1.82 (0.97–3.40)	0.06
200–399	12.80 (3.68–44.58)	<0.001	3.14 (1.19–8.30)	0.021
Time on ART prior enrollment (1 yr increase)	0.90 (0.78–1.05)	0.19	1.11 (1.01–1.22)	0.03
ART regimen on enrollment			
First Line ART	Reference		Reference	
Second line ART	1.80 (0.34–9.66)	0.49	3.94 (1.58–9.84)	0.003
Model type				
Facility AC	Reference		Reference	
Community AC	0.35 (0.04–2.94)	0.33	0.73 (0.14–3.97)	0.72
CAG	1.04 (0.19–5.74)	0.97	1.65 (0.43–6.34)	0.46
SFLA	0.02 (0.01–0.05)	<0.001	0.76 (0.34–1.70)	0.50
PuP	0.01 (0.01–0.04)	<0.001	0.41 (0.22–0.77)	0.005
Year of DMOC enrollment^3^	0.37 (0.32–0.43)	<0.001	0.38 (0.32–0.46)	<0.001

^1^ Weibull model with adjustment for clustering at health center and an individual levels

^2^ Last known measure prior DMOC enrollment

^3^ Year of enrollment into a DMOC model, 2012–2013 as a reference, 1 year increase

Abbreviations: DMOC, differentiated model of HIV care; CAG, community ART group; AC, adherence club; SFLA, spaced and fast lane appointment model; PuP, decentralized medication delivery at community pick up points; SoC, standard of care; ART, antiretroviral therapy; VL, viral load; HR, hazard ratio; aHR, adjusted hazard ratio; CI, confidence interval

## Discussion

Our results demonstrate the effectiveness of an HIV service delivery program offering five different DMOC implemented at scale in a large public sector cohort in KwaZulu-Natal, South Africa. In our study, 36-months outcomes among DMOC patients showed sustained retention on ART and VL suppression.

High retention on ART and VL suppression among clinically stable patients in our study, irrespective of care model, agree with existing empirical evidence [7,18,28–31]. Overall retention on ART among DMOC patients at 12, 24 and 36 months was 96%, 93%, 90%. Similar findings were reported in South Africa for AC and SFLA [30]. Prevalence of VL rebound (≥1000 copies/ml) and confirmed VF was low among patients in all care models. In our study prevalence of VF was ≤1% in DMOC at the end of follow up, which agrees with evidence that ART experienced and clinically stable patients sustain VL suppression in the long term [32]. Except for the early study period (2012–2015), VL suppression <1000 copies/mL was above 95% in all DMOC models at 12 and 24 months, similar to findings of others [18].

The cumulative attrition from DMOC was 27%, largely due to return to SoC (Table 1). The highest rate of attrition from DMOC was observed during the initial 12 months in a DMOC model (15.9 per 100 person-years in the first year), and was 3-times lower in the following years (i.e., 5.2 per 100 person-years during the third year of follow up). Nevertheless, disengagement from a DMOC model has not resulted in higher attrition from ART. These results echo findings from Swaziland [33] and South Africa [29,30], which show ≥15% disengagement from a DMOC model within the initial 12 months, whilst continuing on ART.

The findings also highlight challenges with rapid scale up of PuP and SFLA in our study area. Higher attrition from DMOC was observed among those enrolled during 2016–2018 as compared to the earlier period. Compared to facility AC, PuP model was associated with a higher risk of all cause attrition from DMOC (aHR = 1.21, *p* = 0.02) and from ART (aHR = 1.25, *p* = 0.03). Although we did not explore reasons for return to SoC clinic in this study, the national guidelines [21] stipulate return to SoC for medical or administrative reasons, or they may return for any personal reason including conflicts within a group [33].

Qualitative studies on CCMDD roll out identified positive attitudes and acceptability of community PuP among stakeholders. Nevertheless, several barriers were also evident such as inadequate infrastructure, errors in medication packaging and tracking and over capacity of community pick up points [34,35]. Inflexible pick up hours, rigid CCMDD rules, and concerns over stigma and discrimination outside HIV clinics were among patients’ concern [34,36]. These experiences were documented in townships in KwaZulu Natal [34,35] and may be relevant to our study population as factors that may have contributed to attrition among PuP participants. The fragmented CCMDD monitoring and evaluation system was a challenge in our study, as was documented by others in South Africa [22]. Information on medication pick-ups from private providers was not always reaching patients’ records at health centers [31,34], what might have resulted in misclassification of some patients as LFTU.

Predictors of attrition from DMOC, and from ART, were male gender, younger age (especially 18–29 years), shorter duration on ART prior the enrollment. Young adults were also at increased risk of viral load rebound. These baseline characteristics were found to be significant risk factors for sub-optimal outcomes in other studies [4,7,37,38], and are not limited to DMOC [39–41]. Poor outcomes among young adults and men are one of the long-standing challenges facing HIV programs in our study area [40], and broadly in the region [39]. Fox et al. [31] in a large cluster-randomized evaluation of AC vs standard care shown improved outcomes among men. In our setting these benefits were not evident, men were more likely to disengage from DMOC and from ART. Out-migration from rural KwaZulu-Natal, our study area, for employment and other opportunities may explain these differences. Young adults, and men of working age, frequently travel for opportunities in urban centers (e.g. Durban), without formal transfer-out, thus might have been documented as LTFU.

Our study is among few in the Southern Africa region to provide insights on the importance of LLV and monitoring of patients who are on second line ART in the context of expanding DMOC programs. Low level viremia (>50–399 copies/mL) was associated with increased hazards of VL rebound and attrition from ART, as compared to virological suppression <50 copies/mL. This is in line with findings from a number of settings [42,43] including South Africa [44], and a regional African cohort Study [45], which demonstrated that LLV was associated with a subsequent virological failure. In turn, determinants of LLV were shown to be associated with sub-optimal ART adherence, and history of prior VF including those on the second line ART regimens [46,47]. Although the proportion of those enrolled into DMOC on second line was 5% in our study, they were at increased hazards of attrition from DMOC and VL rebound. Patients on PI-based second line ART regimens may require tailored adherence counseling and clinical monitoring, as recent meta-analyses [48,49] of studies in sub-Sahara Africa have demonstrated that patients on PI-based second line ART are at higher risk of persistent high viral loads or repeat virological failure after initial suppression.

Adoption of CCMDD into AGL has significantly expanded access to ART and treatment of other chronic conditions in South Africa [22]. In our service area, between 2012 and 2018, the proportion of enrolled into DMOC increased from 11.3% to 55.3% among eligible patients [50]. According to CCMDD evaluation in the eight provinces, including KwaZulu Natal, in 2016–2019 there had been an 8-fold increase in CCMDD registrations; 35% of active CCMDD participants were receiving ART at PuP, 52% through SFLA, and 13% in outreach and adherence clubs [22]. In contrast, a relatively higher proportion of patients in our setting were receiving care in AC or CAGs. As of last DMOC visit: 26% received care AC, 2% in CAGs, 30% in SFLA, and 42% in PuP. Contextual factors may have contributed to these differences, among notable are earlier investments by MSF into piloting and implementation of AC and CAGs [19,51].

Roll out of the five DMOC types may have offered many patients some flexibility in choosing a model that better meets their needs. Other studies in South Africa shown that patients were offered an opportunity to choose a preferred DMOC model [52,53]. Despite increased access to PuP and SFLA, many eligible patients in our setting opted to continue with AC or community groups. PuP participants in our study were of younger age, what agrees with findings of an observational study in KwaZulu Natal [54], who shown that uptake of community CCMDD sites was associated with younger age, along with factors such as full time employment, and no self-perceived barriers to care and high self-efficacy.

At the same time, our study identifies health system limits in adoption of DMOC. Implementation of DMOC types varied across ART centers, with apparent preferences for individual (PuP) and facility-based models (facility AC, SFLA) over community-based models (CAG, community AC). We did not conduct evaluation of how adoption of different models took place in participating clinics. Emerging evidence from other settings suggest that adoption of DMOC models at facility levels was often constrained due to complexities of managing group models (e,g. CAG, AC), requirement of additional human resources, competing priorities for community workers, gaps in pharmacy chain, and cost and cost-effectiveness considerations [55,56].

Our study highlights the challenges with differentiating clinically stable patients, and importance of monitoring clinical status over time. Indeed, an estimated 13% of patients enrolled in DMOC in our area were not eligible at the time of enrollment (excluded from this analysis). Clinical stability is a transient concept, depends on defining criteria (e.g. VL or CD4, or both) [55], and can change over time due to lapses in treatment uptake, or relapse of clinical instability [57].

The strengths of this study are the large sample size and long duration of follow up. Although the findings of this study are encouraging, we conclude with caution due to limitations inherent to the observational study design. In this study, we used the routine data collection system TIER.net, and did not account for silent transfers, which may be misclassified as LTFU, leading to underestimates of retention in DMOC and on ART [58,59]. Nevertheless, we invested a significant amount of resources to verify last visits of DMOC patients by reviewing relevant registers at the clinics and the CCMDD registers. Lastly, although we investigated sustainability of DMOC we did not assess its essential components such cost-effectiveness and responsiveness to patients’ needs.

## Conclusions

In conclusion, this study adds to the evidence that DMOCs shown sustained retention in care and viral suppression, while decreasing demands on patients and clinical staff at ART clinics. The study also shows that a wide offer of DMOCs can be effectively operated in a public sector setting in South Africa. Further evaluations are necessary to assess cost-effectives of DMOC models in comparison with the standard of care at clinics.

## Supporting information

S1 File Adaptation of TIER.net for capture of DMOC visits at MSF-supported ART clinics. (DOCX)
