# Correction: Retention on ART and viral suppression among patients in alternative models of differentiated HIV service delivery in KwaZulu-Natal, South Africa

**DOI:** 10.1371/journal.pgph.0003038

**Published:** 2024-03-08

**Authors:** Altynay Shigayeva, Ntombi Gcwensa, Celiwe Dlamini Ndlovu, Nosicelo Ntumase, Scelinhlanhla Sabela, Liesbet Ohler, Laura Trivino-Duran, Ellie Ford Kamara, Khanyo Hlophe, Petros Isaakidis, Gilles Van Cutsem

There are errors in the Funding and Competing Interests statements. The correct statements are:

**Funding:** Médecins Sans Frontières (MSF) provided support for this study in the form of salaries for AS, NG, CDN, NN, SS, LO, LTD, EFK, PI, and GVC. The specific roles of these authors are articulated in the ‘author contributions’ section. Research conducted by MSF employees is done so within the scope of their job specification. No other forms of support were provided specifically for the research. MSF was involved in study design, data collection, analysis, decision to publish and preparation of the manuscript.

**Competing Interests:** The authors have read the journal’s policy and have the following competing interests to declare: Médecins Sans Frontières (MSF) provided support in the form of salaries for AS, NG, CDN, NN, SS, LO, LTD, EFK, PI, and GVC. This does not alter our adherence to *PLOS Global Public Health* policies on sharing data and materials. There are no patents, products in development, or marketed products associated with this research.

## References

[1] Shigayeva A, Gcwensa N, Ndlovu CD, Ntumase N, Sabela S, Ohler L, et al. (2022) Retention on ART and viral suppression among patients in alternative models of differentiated HIV service delivery in KwaZulu-Natal, South Africa. PLOS Glob Public Health 2(12): e0000336. 10.1371/journal.pgph.0000336 36962695 PMC10021436

