# Supplementary material for: Retention on ART and viral suppression among patients in alternative models of differentiated HIV service delivery in KwaZulu-Natal, South Africa
Source: PLOS Glob Public Health. 2022 Dec 14;2(12):e0000336. doi: 10.1371/journal.pgph.0000336 (PMC10021436; doi:10.1371/journal.pgph.0000336)

**Adaptation of TIER.net for capture of DMOC visits at MSF-supported ART clinics**

TIER.net is the national data system for monitoring of ART and tuberculosis treatment. MSF provided technical assistance with data capture, and monitoring of the data quality.

MSF assisted health centers to adapt data capture in TIER.net, in order to facilitate monitoring of DMOC visits. The field *sub-clinic* (header “adherence club” in the enclosed screenshot) in the Visit module was used to indicate if a visit was a DMOC visit. Originally, the field was set up for the Adherence clubs then expanded to include other DMOC types. The system was set up as follow:

- Each DMOC at a facility had a reference ID, with a facility number embedded. For example 1012-CAG1, 1012-CAG2, 1012-AC club 1, 1012-SFLA
- Existing CCMDD pick points in a community were also assigned a distinct ID
- The drop down list for *sub-clinic* variable was updated regularly
- Data captures were trained how to enter the DMOC visits data from registries and log books at the facilities, CCMDD pick notes in the patient’ files.

This sub-clinic field was important source of information during the assessment if a participant left or remained in a particular model. The check box “CCMDD” that was introduced in 2018 into TIER.net. The updates were dependent on availability of pick up information from CCMDD providers.


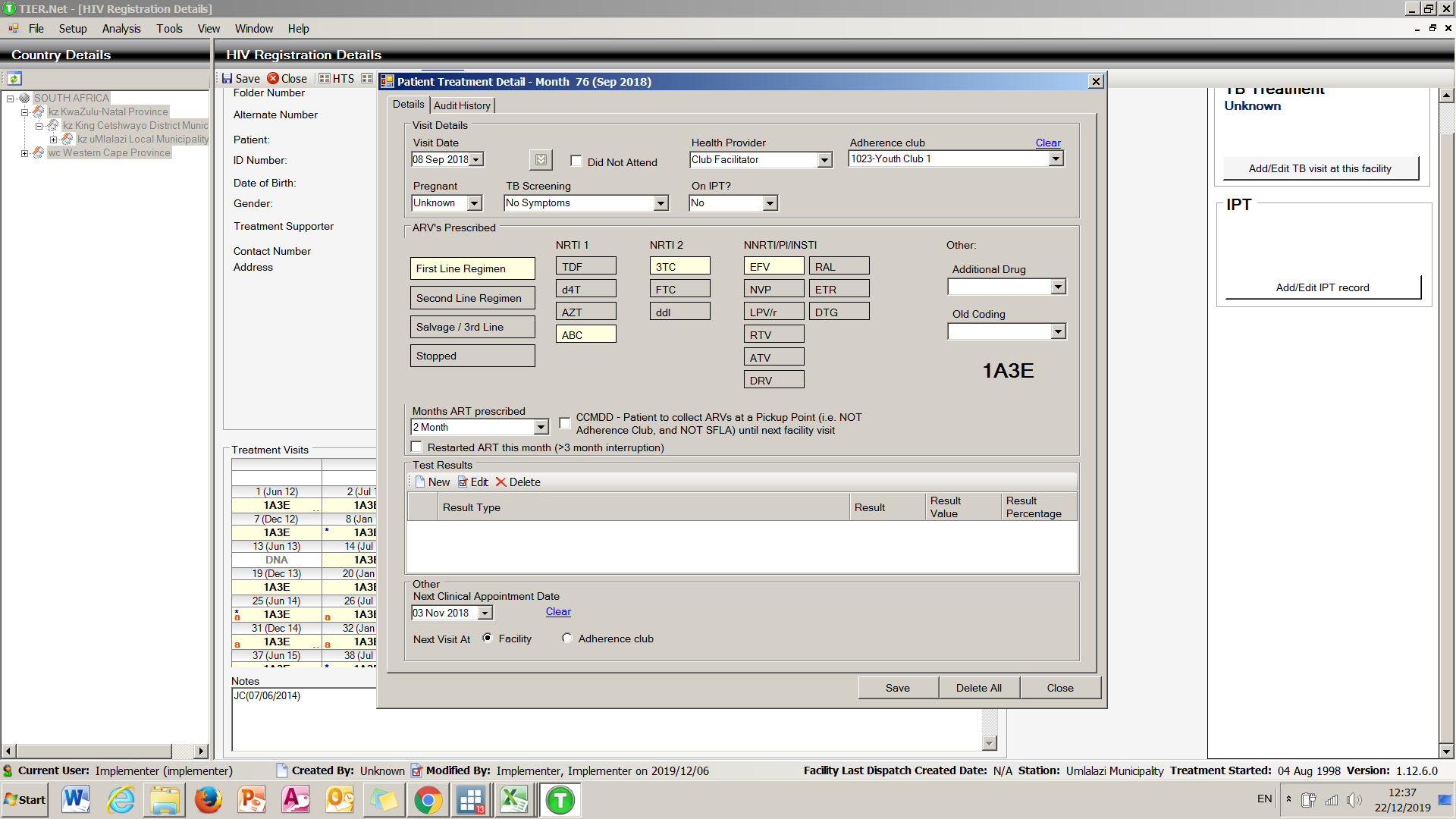

Supplement: S1 File — (DOCX) [file pgph.0000336.s001.docx]
